# Clinical Performance of Immediately Restored, Surface Modified Two‐Piece Zirconia Implants. A Prospective Case Series

**DOI:** 10.1111/clr.70034

**Published:** 2025-08-27

**Authors:** G. Trimpou, A. Begić, K. Obreja, A. Montada, P. Parvini, F. Schwarz

**Affiliations:** ^1^ Department of Oral Surgery and Implantology Goethe University, Carolinum Frankfurt am Main Germany

**Keywords:** clinical study, immediate restoration, zirconia implants

## Abstract

**Purpose:**

To evaluate the short‐term success and survival rates of immediately restored surface modified two‐piece zirconia implants (CL).

**Material and Methods:**

A total of 23 patients had received CL implants (*n* = 26) for single‐tooth replacements in the anterior and posterior maxilla and mandible at healed extraction sites. Immediate implant restoration was accomplished if an insertion torque ≥ 30 Ncm was reached (Type 3–4 A protocol). Final occlusally screw‐retained crowns were provided after 3 months of healing.

Implant survival and success (i.e., bleeding on probing—BOP, probing pocket depth—PD, mucosal recession—MR) were assessed at 6 and 12 months following final restoration.

Patient‐reported outcomes were evaluated at 6 and 12 months.

**Results:**

Five implants (*n* = 5 patients) could not be immediately restored, and four implants were lost during follow‐up. The survival rates amounted to 80%. For the final analysis, 16 patients with a total of 16 implants were evaluated. Minor changes to baseline (final restoration) were noted at 6 and 12 months for mean BOP (−3.3% ± 13.14%; 8.3% ± 16.9%), PD (−0.15 ± 0.48 mm; 0.08 ± 0.48 mm), and MR (0.0 ± 0.0 mm; 0.0 ± 0.0 mm) values. Mechanical and technical complications were not observed. Patients expressed an overall high satisfaction at 6 and 12 months.

**Conclusions:**

The presented immediate restoration protocol may be associated with an increased risk for early implant losses.

## 1 Introduction

Nowadays, ceramic implants have become a viable alternative to titanium implants for the support of both single crowns and overdentures. This was evidenced by comparable survival rates ranging from 67.6% to 93.3% for zirconia and from 66.7% to 100% for titanium implants, respectively (Padhye et al. 2023). Furthermore, two recent systematic reviews also pointed to high success rates of zirconia implants as indicated by marginal bone levels, peri‐implant tissue health status, and pink esthetic scores (Padhye et al. 2023; Roehling et al. 2023).

Despite these promising outcomes, however, zirconia implants tended to be associated with higher early implant losses within the first year of conventional loading (Padhye et al. 2023).

In this context, it must be realized that in contemporary implant dentistry, there is an ongoing trend towards immediacy, including immediate implant placement and restoration (Cosyn and Blanco 2023). Indeed, the latter protocols have evolved to evidence‐based approaches, particularly for immediate placement and restoration of titanium implants (Donos et al. 2021; Morton et al. 2023; Tonetti et al. 2019).

In contrast, zirconia implants are still more commonly approached by conventional placement and loading protocols (Padhye et al. 2023; Roehling et al. 2023). In previous years, zirconia implants have undergone major surface modifications to optimize bone tissue response and stability, thus potentially allowing for an earlier implant restoration/loading (Depprich et al. 2014). In fact, various surface technologies were associated with an improved osseointegration and removal torque values of zirconia implants, as evidenced in different animal species. This was particularly true for micro‐rough surfaced zirconia implants, which were on a similar level to titanium implants in terms of soft and hard tissue integration potential (Roehling et al. 2019).

The aim of the present prospective observational study was to assess the short‐term survival and success rates of immediately restored, surface‐modified two‐piece zirconia implants.

## 2 Material and Methods

### 2.1 Study Design and Participants

The study protocol was approved by the ethics committee of the Goethe University, Frankfurt, Germany (Approval number: 20‐633) and registered via the Internet Portal of the German Clinical Trials Register (DRKS00022174) on July 1st 2020, and was conducted in accordance with the Declaration of Helsinki, as revised in 2013, for human studies. Each patient was given a detailed description of the study procedures and signed an informed consent before participation. Participants enrolment started on 06/01/2021, and all the surgical procedures were carried out between February 2021 and December 2022. The present reporting considered the STROBE checklist items (von Elm et al. 2014) (Table 1).

**TABLE 1:** (a) Patient characteristics. (b) Reasons for tooth extraction and (c) implant site characteristics.

(a)	
Patient age	50.38 ± 15.13 years (median: 54 years)
Female/male	*n* = 6/10
ASA physical status classes	I (normal healthy) = 11 II (mild systemic disease) = 5
Smoking	none = 12 < 10 cigarettes per day = 4
(b)	
Reason for tooth extraction	Caries = 3 Endodontic lesion = 5 Fracture = 4 Unknown = 4
(c)	
Mucosal thickness	1.37 ± 0.76 mm
Distance implant – adjacent tooth	Mesial = 2.97 ± 1.1 mm Distal = 2.97 ± 1.2 mm
Implant length	8 mm = 5 10 mm = 9 12 mm = 2
Insertion torque	≥ 30 Ncm (*n* = 16)

For this study, a total of 25 patients attending the Department of Oral Surgery and Implantology at the Goethe University Frankfurt, Germany for single tooth replacements in the anterior and/or posterior maxilla and/or mandible were recruited. One patient was excluded due to a screening failure and one patient withdrew participation. Accordingly, a total of 23 patients were included. Three out of 23 patients had received two implants each, thus resulting in a total of 26 implants.

### 2.2 Inclusion Criteria

To be included in the study, the patients had to fulfill all of the following conditions:

1. Patients must have voluntarily signed the informed consent form before any study‐related action.
2. Patients with an age of at least 18 years.
3. Patients being in need of a single tooth restoration in a partially edentulous mandible and/or maxilla: single tooth gaps with sufficient bone for the placement of an implant with a diameter of 4.5 mm (implant neck); adjacent mesial and distal teeth must be natural teeth.
4. Tooth extraction at least 12 weeks prior to implant placement.
5. Opposing dentition must be natural teeth or fixed restorations.
6. Systemically healthy with no contraindications for oral surgical procedures.
7. Willingness to attend all follow‐up visits as defined in the study protocol.

### 2.3 Exclusion Criteria

1. Smokers > 10 cigarettes per day or cigar equivalents or chewing tobacco.
2. Systemic diseases that would interfere with implant therapy (e.g., uncontrolled diabetes, HbA1c > 7%).
3. Uncontrolled para‐functional diseases (bruxism, clenching or grinding of teeth).
4. Conditions or circumstances, in the opinion of the investigator, which would prevent completion of study participation or interfere with analysis of study results, such as history of non‐compliance or unreliability.
5. Pregnant or lactating women.

### 2.4 Clinical Assessment of Primary and Secondary Outcomes

The primary outcomes considered the peri‐implant tissue health status (Tonetti et al. 2023), as evaluated by the clinical assessment of bleeding on probing—BOP, probing pocket depth—PD (measured from the mucosal margin to the bottom of the probeable pocket), and mucosal recession—MR (measured from the crown margin to the mucosal margin).

The clinical measurements were recorded at six sites per implant using a periodontal probe: mesiobuccal (mb), midbuccal (b), distobuccal (db), mesiooral (mo), midoral (o), and distooral (do).

Two‐dimensional radiographs for the assessment of marginal bone levels were not routinely justified according to the German X‐Ray Regulation (Röntgenverordnung) based on the 97/43/EURATOM directive and the German Radiation Protection Ordinance (Strahlenschutzgesetz) based on the 103/2013 Euratom directive. Consequently, radiographs were only taken if clinically justified (i.e., when clinical signs suggested the presence of peri‐implantitis or mechanical complications).

The secondary outcomes included implant survival (i.e., presence of the implant in situ at the 6 and 12 month follow‐up examinations), insertion torque at implant placement, plaque index—PI (Löe 1967), the presence of peri‐implant diseases, mechanical and technical complications as well as patient‐reported outcome measures (PROM's) (Trimpou et al. 2022).

The presence of peri‐implant diseases at 6 and 12 months was evaluated based on established case definitions (Berglundh et al. 2018). Peri‐implant mucositis was defined as the presence of BOP and/or suppuration with or without increased PD, and peri‐implantitis as the presence of BOP and/or suppuration with increased PD and the presence of radiographic bone loss (i.e., baseline to 6 and 12 months) (Berglundh et al. 2018).

Mechanical complications considered all the events affecting the integrity of the implant or of the abutment. Technical complications considered all the events affecting the integrity of the implant‐supported restoration (i.e., crown).

PROM's were assessed at 6 and 12 months by a questionnaire on the (1) prosthesis (i.e., crown) comfort, (2) prosthesis appearance, (3) chewing, (4) tasting ability, (5) prosthesis fit, and (6) overall satisfaction, with scores ranging from 1 (very satisfactory) to 5 (unsatisfactory) (Trimpou et al. 2022). Before the clinical assessments at the 6 and 12‐month follow‐ups, patients completed the designated questionnaires at the clinic without their treating practitioner present.

Primary and secondary outcomes were assessed by three calibrated and experienced investigators (A.B., K.O., G.T.).

### 2.5 Sample Size Calculation

A sample size calculation was not feasible due to insufficient reference data on this immediacy protocol in the existing literature.

### 2.6 Implant and Abutment Materials

For this study, two‐piece CERALOG Plus Hexalobe Implants (diameter implant: 4 mm, diameter implant neck: 4.5 mm) and the corresponding prosthetic and lab components (Camlog Biotechnologies GmbH, Basel, Switzerland) were used. These implants consisted of ultra‐pure yttrium stabilized tetragonal zirconium dioxide (Y‐TZP) manufactured by ceramic injection molding (Beger et al. 2018). The rough surface texture was modified with a nano‐thin coating resulting in Ra values of 1.6 (implant body) and 0.5 (implant neck). For immediate and final restorations, abutments made of polyetheretherketone (PEEK) (CERALOG PEEK abutment, Altatec GmbH, Wimsheim, Germany) and polyetherketoneketone (PEKK) (CERALOG PEKK abutment, Altatec GmbH) were used.

All implants and components were CE‐marked and used within the indications recommended by the manufacturer.

### 2.7 Surgical and Prosthetic Procedures

**FIGURE 1:**
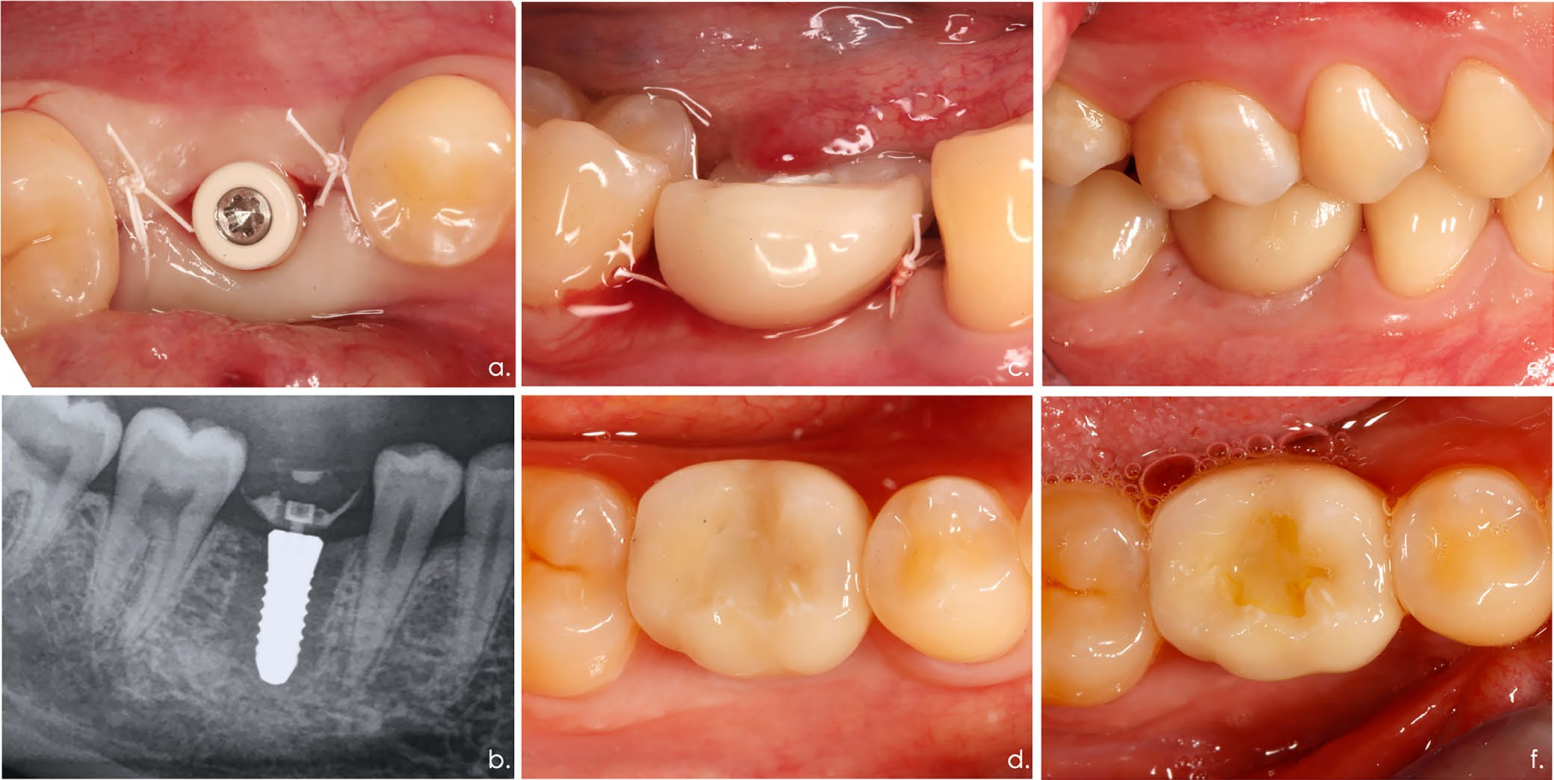
Surgical and prosthetic procedures. (a) Situation following implant placement region 046. (b) In case of an adequate primary implant stability (i.e., insertion torque of ≥ 30 Ncm), a provisional (non‐radiopaque) PEEK abutment was connected (25 Ncm) and a prefabricated‐shell provisional crown provided. Panoramic radiograph taken following immediate restoration. (c) Clinical view indicating that the immediate restoration was in non‐functional occlusion. (d) After 3 months of healing, the definitive occlusally screwed crown was provided. (e) Clinical situation at 6 months. (f) Clinical situation at 12 months.

Mucoperiosteal flaps were elevated at respective sites under local anesthesia (4% articaine plus epinephrine 1:200,000). Following meticulous granulation tissue removal and inspection of the integrity of the alveolar ridge, implant bed preparation was accomplished according to the recommendations given by the manufacturer. The protocol included a round bur followed by a pilot drill and two form drills (S and M). An additional tap was used in bone density classes of 1 and 2 (Lekholm and Zarb 1985). Implant placement considered a distance to the adjacent teeth of at least 1.5 mm and a vertical insertion to the smooth‐rough border, thus resulting in a supracrestal implant position of about 1.2 mm (Figure 1a). Minor bone grafting procedures employing a bovine bone mineral (BioOss, Geistlich Biomaterials, Wolhusen, Switzerland) and a collagen membrane (BioGide, Geistlich Biomaterials) were needed at two implant sites (*n* = 2 patients).

When an insertion torque of 30 Ncm was obtained, intraoral scans were taken (Cerec Omnicam; Dentsply SironaM, Bensheim, Germany) to assess the implant position (CERALOG Scanbody, Altatec GmbH) and to register the opposing jaw (Table 1).

Subsequently, a provisional PEEK abutment was connected (25 Ncm) and a prefabricated‐shell provisional crown (Vita Physiodens, VITA Zahnfabrik H. Rauter GmbH & Co. KG, Bad Säckingen, Germany) was filled up with acrylic (Pro TempTM 3 Garant, 3 M ESPE, 3 M Deutschland GmbH, Neuss, Germany) and adapted. The occlusal screwed temporary was inserted with a torque of 15 Ncm without occlusal contact (Figure 1b,c). The occlusion was visually checked with the use of an articulation film. All patients were instructed to adhere to a soft diet for a period of 12 weeks. In addition, all patients were instructed in the usual mechanical oral hygiene procedures.

After 3 months of healing, the definitive occlusally screwed crown (Katana Zirconia Block, CZR Press, Kuraray Noritake Dental Inc.) with full occlusion delivered on a PEKK abutment (Panavia 5, Kuraray Noritake Dental Inc., Tokyo, Japan) was connected (25 Ncm) (Figure 1d).

### 2.8 Postoperative Care

Postoperative care involved professional supramucosal−/gingival implant and tooth cleaning as well as an oral hygiene reinforcement at 6 and 12 months.

### 2.9 Statistical Analysis

The statistical analysis of the pseudonymised data sets was accomplished using a commercially available software program (IBM SPSS Statistics 27.0, IBM Corp., Armonk, NY, USA).

Mean values, standard deviations, medians, 95% confidence intervals (CI) and frequency distributions were calculated for all clinical parameters.

For the changes in clinical parameters (i.e., baseline—6 and 12 months), interquartile ranges (IQR) were also calculated. Data were checked for normality using the Shapiro–Wilk test.

Changes in mean BOP, PD, and MR values at 6 and 12 months relative to baseline were assessed using the Friedman test.

The alpha error was set at 0.05.

## 3 Results

An adequate primary implant stability (i.e., insertion torque ≥ 30 Ncm) could not be obtained a total of five patients. These implants (*n* = 5) were allocated to a submerged healing procedure, successfully restored and maintained over 12 months, but excluded from this per‐protocol analysis. Three of these patients had received two implants each, with the second implant showing adequate primary stability. A total of four implants in four patients were explanted due to implant loosening at 6, 9, 11, and 20 weeks after the immediate restoration. One patient was lost to follow‐up. For the final analysis, 16 patients (10 males, 6 females, mean age: 50.38 ± 15.13 years) with a total of 16 implants were evaluated.

### 3.1 Clinical Measurements

Mean and median PI, BOP, PD, and MR values measured at 6 and 12 months are summarized in Table 2.

**TABLE 2:** (a) Clinical parameters measured at Baseline (i.e., final restoration) (*n* = 16 patients). (b) Clinical parameters measured at 6 months (*n* = 16 patients). (c) Clinical parameters measured at 12 months (*n* = 16 patients).

	BOP	PD	MR	PI
(a)				
Mean	6.67	2.78	0.02	0.00
SD	11.65	0.69	0.07	0.00
Median	0.00	3.00	0.00	0.00
IQR	17.0	0.83	0.00	0.00
95% CI	1.67; 15.0	2.28; 3.28	−0.03; 0.08	0.00; 0.00
(b)				
Mean	16.67	2.35	0.02	0.2
SD	28.32	0.45	0.07	0.31
Median	0.00	2.33	0.00	0.00
IQR	25.0	0.63	0.00	0.42
95% CI	−0.3; 36.7	2.02; 2.67	−0.03; 0.08	−0.02; 0.42
(c)				
Mean	16.67	2.53	0.03	0.03
SD	20.78	0.45	0.10	0.10
Median	8.33	2.66	0.00	0.00
IQR	37.0	0.63	0.00	0.00
95% CI	1.8; 31.5	2.20; 2.86	−0.04; 0.10	−0.04; 0.10

At 6 and 12 months, all patients investigated were associated with a good level of oral hygiene, as indicated by median PI scores of 0.00, respectively (Figure 1e,f) (Table 2). Median BOP scores at 6 and 12 months amounted to 0.00% and 8.33% with only minor mean changes to baseline of −3.3% ± 13.4% and 8.3% ± 16.9%, respectively (*p* = 0.734, Friedman test) (Tables 2 and 3). Median PD scores at 6 and 12 months were 2.35 and 2.66 mm. The respective changes to baseline were minor and amounted to −0.15 ± 0.48 mm and 0.08 ± 0.48 mm, respectively (*p* = 0.032, Friedman test) (Tables 2 and 3). After 6 and 12 months, mean MR values remained unchanged (*p* = 0.368, Friedman test) (Tables 2 and 3).

**TABLE 3:** (a) Changes (d) in clinical parameters between baseline and 6 months (*n* = 16 patients). (b) Changes (d) in clinical parameters between baseline and 12 months (*n* = 16 patients).

	dBOP	dPD	dMR	dPI
(a)				
Mean	−3.3 ± 13.14	−0.15 ± 0.48	0.0 ± 0.0	0.03 ± 0.10
Median	0.0	−0.16	0.0	0.00
IQR	4.0	0.58	0.00	0.00
95% CI	−12.7; 6.07	−0.49; 0.19	0.0; 0.0	−0.04; 0.10
(b)				
Mean	8.3 ± 16.19	0.08 ± 0.48	0.0 ± 0.0	0.05 ± 0.11
Median	0.0	0.25	0.0	0.00
IQR	21.0	0.54	0.00	0.04
95% CI	−3.25; 19.92	−0.26; 0.43	0.0; 0.0	−0.03; 0.13

### 3.2 Implant Survival

At 6 and 12 months, the implant survival amounted to 80.0%, respectively.

### 3.3 Incidence of Peri‐Implant Diseases

The incidence of peri‐implant diseases at 6 and 12 months is summarized in Table 4. According to the given case definitions, the incidence of peri‐implant mucositis and peri‐implantitis at both patient and implant levels amounted to 31.25% and 0.0% at 6 months and to 68.75% and 0.0%, respectively.

**TABLE 4:** Incidence of peri‐implant diseases at 6 and 12 months.

			Diagnosis			Total
			0	1	2	
	6 Months	Count	11	5	0	16
		%	68.75%	31.25%	0.0%	100.0%
	12 Months	Count	5	11	0	16
		%	31.25%	68.75%	0.0%	100.0%

*Note:* Diagnosis: 0 = peri‐implant health; 1 = peri‐implant mucositis; 2 = peri‐implantitis.

### 3.4 Incidence of Technical and Mechanical Complication

Throughout the observation period of 12 months, no mechanical or technical complications were observed.

### 3.5 PROM's

The mean values for the evaluated questionnaire items (1, 2, 4, 5 and 6) at baseline, 6 months, and 12 months are presented in Table 5. The maximum for all items was 2 at all three time points.

**TABLE 5:** PROM's.

	Baseline	6 months	12 months
Prosthesis comfort			
Mean	1.40	1.00	1.10
SD	0.51	0.00	0.31
Prosthesis appearance			
Mean	1.40	1.00	1.20
SD	0.51	0.00	0.42
Chewing ability			
Mean		1.10	1.10
SD		0.31	0.31
Tasting ability			
Mean	1.20	1.10	1.10
SD	0.42	0.31	0.31
Prosthesis fit			
Mean	1.10	1.40	1.20
SD	0.31	0.51	0.42
Overall satisfication			
Mean	1.20	1.20	1.10
SD	0.42	0.42	0.31

## 4 Discussion

The aim of the present prospective observational case study was to evaluate the short‐term survival and success rates of immediately restored, surface modified two‐piece zirconia implants. This immediacy concept considered an established restoration protocol for titanium implants (Parvini et al. 2020).

When evaluating the present analysis, it was noted that this immediacy concept could not be applied in a total of five patients (*n* = 5 implants), as a consequence of an inadequate primary implant stability (i.e., < 30 Ncm). In this context, it must be emphasized that the insertion torque is influenced by various factors (e.g., bone quality, implant design, implant length and diameter, location) but not necessarily by the implant material (i.e., Y‐TZP) (Monje et al. 2014, 2019). Moreover, the existing literature is still inconclusive regarding the influence of the insertion torque and the survival rates of titanium implants (Darriba et al. 2023; de Douglas Oliveira et al. 2016). Indeed, a systematic review and meta‐analysis pointed to similar survival rates for insertion torques of either ≤ 35 Ncm or > 35 Ncm (96% vs. 92%). However, it was noted that an insertion torque > 35 Ncm was associated with higher survival rates than lower insertion torque values when single implant restorations were evaluated (Darriba et al. 2023). Accordingly, the present study protocol defined an insertion torque of ≥ 30 Ncm as a prerequisite for this immediacy concept. Nevertheless, it still remains unclear to what extent the aforementioned meta‐analysis may also be applied to Y‐TZP implants.

When further evaluating the present survival rates, it becomes apparent that these are on a level lower than the respective values reported for immediately restored titanium implants (Darriba et al. 2023; de Douglas Oliveira et al. 2016). At the time being, data on the immediate restoration of Y‐TZP implants are very limited (Balmer et al. 2020; Borgonovo et al. 2021; Kohal et al. 2023) In particular, Borgonovo et al. (2021) provided the final reconstructions after 24 weeks and reported on an implant survival of 100% over a period of 10 years (12 patients, 29 implants). Balmer et al. (2020) provided the final restorations after 16 weeks in the maxilla and after 8 weeks in the mandible. The survival rate at 5 years amounted to 98.4%. However, merely 53 out of 60 patients, corresponding to 63 out of 71 implants, could be evaluated.

The survival rates as reported in both studies (Balmer et al. 2020; Borgonovo et al. 2021) are obviously higher than those noted in the present study. This might be explained by various factors. First, Borgonovo et al. (2021) reported that “the provisional restorations cemented on single implants were stabilized with composite wings to the adjacent teeth”, thus pointing to a different immediate restoration concept. Secondly, Balmer et al. (2020) also included patients that were in need of “two terminal implants for a 3‐unit implant‐supported bridge”. Moreover, both studies commonly employed longer implants and did not report on insertion torque values (Balmer et al. 2020; Borgonovo et al. 2021).

In Kohal et al. (2023), one‐piece Y‐TZP single tooth implants with immediate provisionalization were definitively restored after a healing period of 2 months in the mandible and 4 months in the maxilla. In comparison to the present study, the slightly lower survival rate at the 5‐year follow‐up amounted to 78.2%. Howeever, Kohal et al. (2023) reported insertion torque lower than 35 Ncm in 4 cases, 35 to 45 Ncm in 38 cases, and greater than 45 Ncm in 17 cases and included both immediate and delayed implant insertion (Kohal et al. 2023).

Accordingly, the latter studies can hardly be compared with the current analysis. This is further impeded by some methodological drawbacks associated with the current study, such as the lack of a sample size calculation, the limited number of participants, as well as the lack of a control group.

When further evaluating the present data set, it was also noted that after the short‐term follow‐up period of 12 months, the presented immediacy concepts was associated with non‐significant changes in mean BOP (8.3% ± 16.9%), PD (0.08 ± 0.48 mm), and MR (0.0 ± 0.0 mm) values. Furthermore, there was an absence of any mechanical or technical complications, which may also have contributed to the high PROM scores observed at 6 and 12 months. The present frequency of peri‐implant mucositis is within the range of previously published epidemiological data on titanium implants (i.e., 43%; CI: 32%–54%) (Derks and Tomasi 2015). However, the noted trend towards a higher incidence of peri‐implant mucositis lesions at Y‐TZP implants at 12 months (64.1% ± 27.2%) was also confirmed in a previous clinical study (Becker et al. 2017).

Finally, an important factor to consider is the manufacturing process of the Y‐TZP implants used in this clinical study.

Using the Ceramic Injection Molding (CIM) technique, both the implant's macrogeometry and surface topography are established prior to sintering and Hot Isostatic Pressing (HIP). This process results in the formation of evenly distributed surface droplets, as previously described by Beger et al. (2018). Additionally, surface characterization has revealed a broad range of roughness values, with pronounced surface irregularities even in areas subjected to machining (Beger et al. 2018).

A recent prospective clinical study on two‐piece zirconia implants fabricated using CIM reported a low 12‐month implant survival rate of 60.9% (Jennes et al. 2025). In that study, two implants failed due to lack of osseointegration at the time of reentry, four exhibited mobility after provisional or definitive prosthetic loading, and three fractured. Although the surgical and prosthetic protocols employed by Jennes et al. (2025) differ from those applied in the present study, a potential contribution of CIM‐related surface characteristics to the reported implant failures cannot be ruled out.

Additionally, experimental research has demonstrated that cyclic loading, combined with the aging of injection‐molded two‐piece zirconia implants with PEKK abutments, significantly increases phase transformation on both the internal and external implant surfaces. However, the monoclinic phase content remains consistently distributed, regardless of its location on the roughened surface or the smooth transgingival area (*p* = 0.931, *η* ^2^ = 0.001) (Zhang et al. 2020). Additionally, the mean fracture load of zirconia implants significantly decreases following cyclic loading and aging (Zhang et al. 2020). Despite this, no implant fractures were observed in the present study. Nevertheless, further research is necessary to validate the long‐term reliability of injection‐molded two‐piece zirconia implants with PEKK abutments.

Within the limitations of the present study, it was concluded that the presented immediate restoration protocol of the surface modified two‐piece zirconia implants may be associated with an increased risk for early implant losses.

## Author Contributions

**G. Trimpou:** conceptualization, funding acquisition, formal analysis, data curation, writing – original draft, writing – review and editing. **A. Begić:** data curation, formal analysis, writing – original draft, writing – review and editing. **K. Obreja:** data curation, formal analysis, writing – review and editing. **A. Montada:** data curation, formal analysis, writing – review and editing. **P. Parvini:** writing – review and editing. **F. Schwarz:** conceptualization, funding acquisition, writing – original draft.

## Conflicts of Interest

Frank Schwarz and Georgia Trimpou have received a research grant for the present study from the Oral Reconstruction Foundation. Frank Schwarz has received lecture fees from the Oral Reconstruction Foundation. The authors declare no conflicts of interest.

## Data Availability

The data that support the findings of this study are available from the corresponding author upon reasonable request.
